# Supplementary material for: Interactome of the HIV-1 proteome and human host RNA
Source: EMBO Rep. 2024 Aug 9;25(9):4078–90. doi: 10.1038/s44319-024-00222-6 (PMC11387401; doi:10.1038/s44319-024-00222-6)
Supplement: Supplementary file 1 — Appendix [file 44319_2024_222_MOESM1_ESM.pdf]

Appendix to: Interactome of the HIV-1 proteome and human host RNA

Tinus Schynkel[1], Willem van Snippenberg[1,2], Kimberly Verniers[2], Gwendolyn M. Jang[3,4,5], Nevan J. Krogan[3,4,5], Pieter Mestdagh[2], Linos Vandekerckhove[1†], Wim Trypsteen[1,2†]

[1] HIV Cure Research Center, Department of Internal Medicine and Pediatrics, Ghent University and Ghent University Hospital, Ghent, 9000, Belgium.
[2] OncoRNALab, Center for Medical Genetics (CMGG), Ghent University, Ghent, 9000, Belgium.
[3] Department of Cellular and Molecular Pharmacology, University of California, San Francisco, CA 94158, USA.
[4] Quantitative Biosciences Institute (QBI), University of California, San Francisco, CA 94158, USA
[5] J. David Gladstone Institutes, San Francisco, CA 94158, USA
† These authors contributed equally to this work.
* Correspondence: Linos.Vandekerckhove@UGent.be, Wim.Trypsteen@ugent.be; Tel.: +3293320698

Appendix table of content

Appendix Figure S1: p2-3
Appendix Figure S2: p4
Appendix Figure S3: p5
Appendix Figure S4: p6
Appendix Figure S5: p7
Appendix Figure S6: p8
Appendix Figure S7: p9
Appendix Figure S8: p10
Appendix Figure S9: p11
Appendix Figure S10: p12
Appendix Figure S11: p13
Appendix Figure S12: p14

Appendix Table S1: p15
Appendix Table S2: p16
Appendix Table S3: p17
Appendix Table S4: p18
Appendix Table S5: p19
Appendix Table S6: p20-25
Appendix Table S7: p26
Appendix Table S8: p27-28

Appendix Figure S1

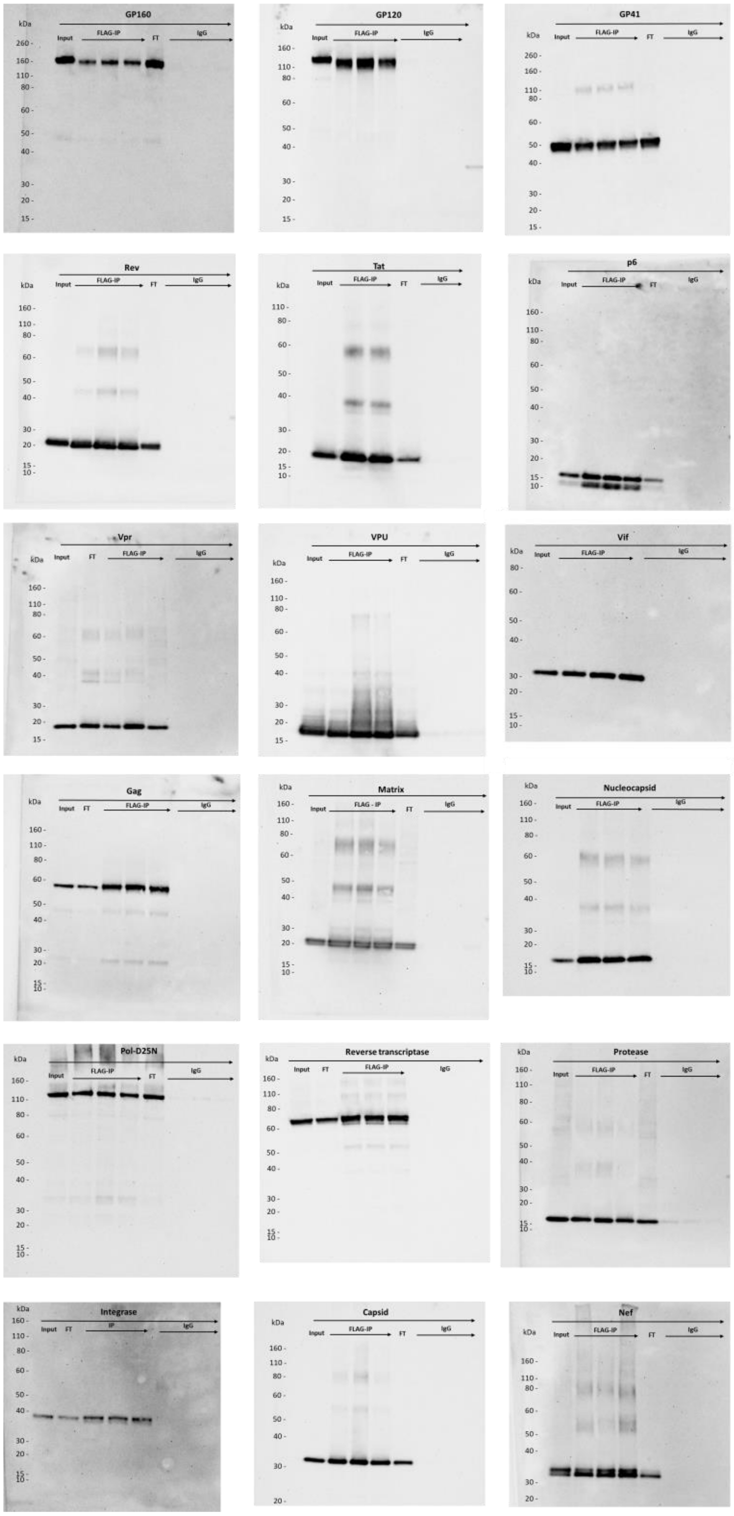

Appendix Figure S1: Western blot confirmation of HIV-1 protein pulldown during FLAG-based RIPseq assay in Jurkat cells. Jurkat T-Rex cells expressing a single 2xStrepTagII-TEV-3xFLAG-tagged HIV-1 protein upon doxycycline induction were lysed, homogenized, and immunoprecipitated with anti-FLAG antibody and magnetic Dynabeads. 5 μL of the washed beads were boiled and analyzed by 4-12% SDS-PAGE. FT: lysate flow-through of immunoprecipitation. IgG: immunoprecipitation with mouse IgG antibody as background control.

Appendix Figure S2

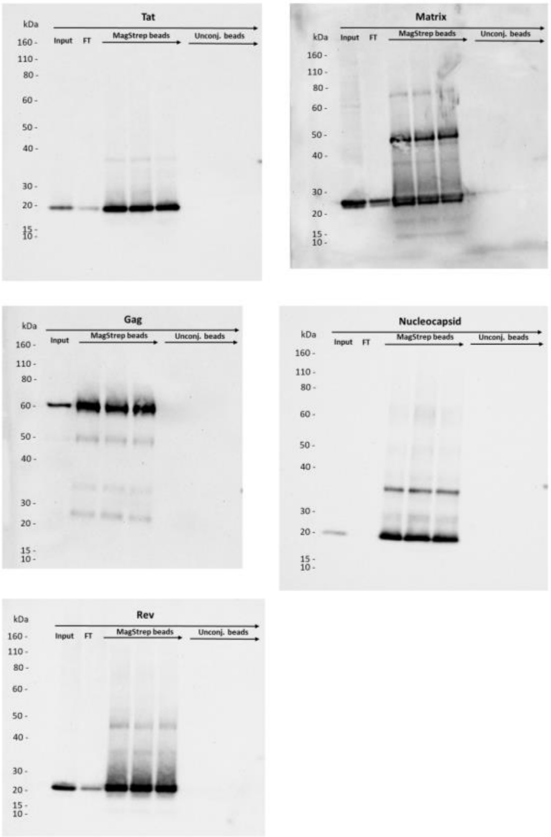

Appendix Figure S2: Western blot confirmation of HIV-1 protein pulldown during Strep-based RIPseq assay in Jurkat cells. Jurkat T-Rex cells expressing a single 2xStrepTagII-TEV-3xFLAG-tagged HIV-1 protein upon doxycycline induction were lysed, homogenized, and immunoprecipitated with MagStrep “type3” XT beads. 5 µL of the washed beads were boiled and analyzed by 4-12% SDS-PAGE. FT: lysate flow-through of immunoprecipitation. Unconj. Beads: immunoprecipitation with unconjugated XT beads as background control.

Appendix Figure S3

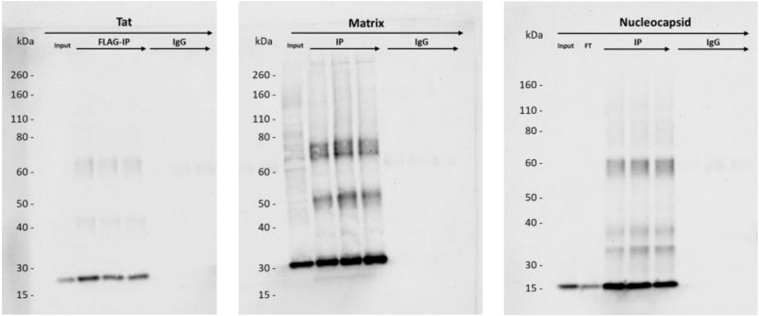

Appendix Figure S3: Western blot confirmation of HIV-1 protein pulldown during FLAG-based RIPseq assay in SupT1 cells. SupT1 cells constitutively expressing a single 2xStrepTagII-TEV-3xFLAG-tagged HIV-1 protein upon doxycycline induction were lysed, homogenized, and immunoprecipitated with anti-FLAG antibody and magnetic Dynabeads. 5 μL of the washed beads were boiled and analyzed by 4-12% SDS-PAGE. FT: lysate flow-through of immunoprecipitation. IgG: immunoprecipitation with mouse IgG antibody as background control.

Appendix Figure S4

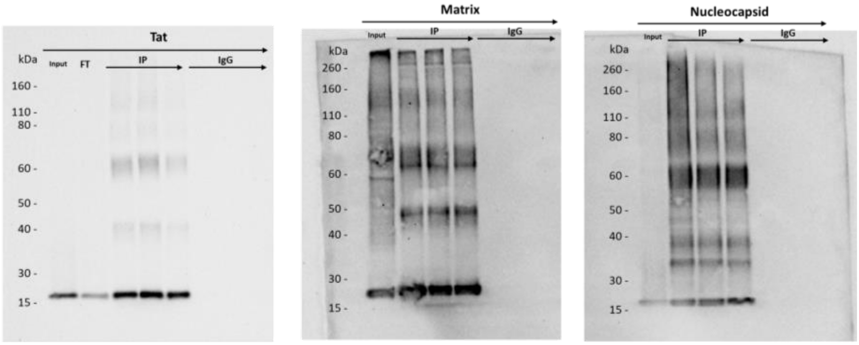

Appendix Figure S4: Western blot confirmation of HIV-1 protein pulldown during FLAG-based RIPseq assay in HIV-1 infected SupT1 cells. SupT1 cells constitutively expressing a single 2xStrepTagII-TEV-3xFLAG-tagged HIV-1 protein upon doxycycline induction were infected with NL4.3 HIV-1 virus for 24h, lysed, homogenized, and immunoprecipitated with anti-FLAG antibody and magnetic Dynabeads. 5 μL of the washed beads were boiled and analyzed by 4-12% SDS-PAGE. FT: lysate flow-through of immunoprecipitation. IgG: immunoprecipitation with mouse IgG antibody as background control.

Appendix Figure S5

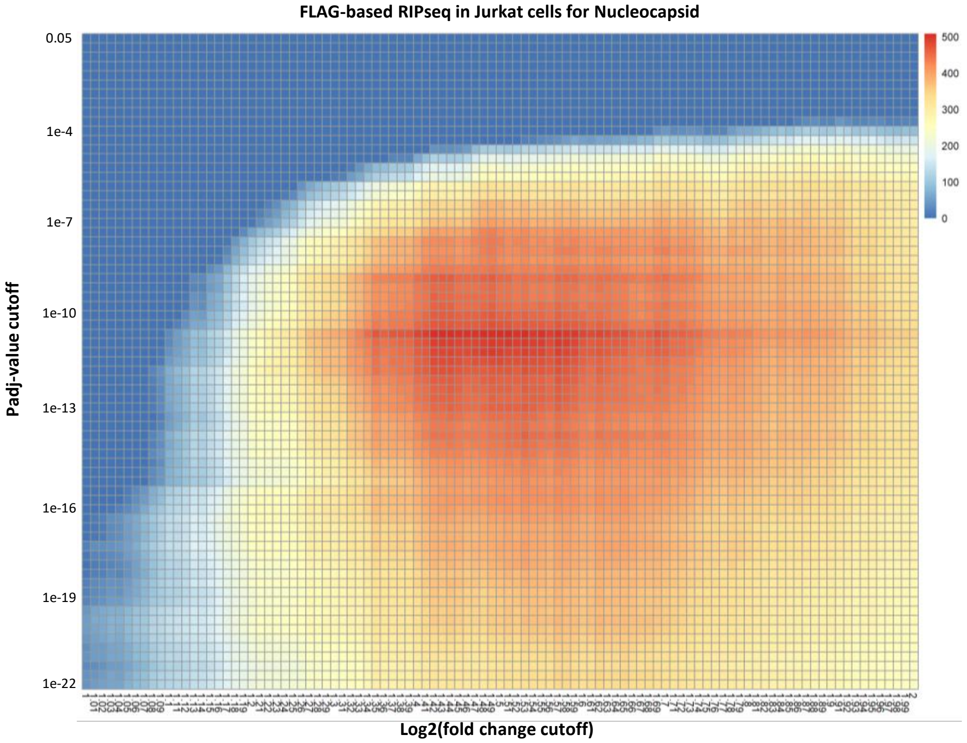

Appendix Figure S5: Example of foldchange cutoff and Padj-cutoff optimization. The foldchange cutoff and Padj-cutoff to accept a gene with a set Padj-value and foldchange as RNA interaction partner of an HIV-1 protein were individually determined per protein by optimizing the signal to background proportion, through maximizing a RIPseq score. This heatmap show an example of this optimization: for the FLAG-based RIPseq of Nucleocapsid in Jurkat cells. The heatmap shows the RIPseq score for every Padj cutoff value and Log2FC-value. For this RIPseq experiment the optimal values were eventually set as Padj cutoff = 10e-11 and Log2FC-cutoff = 1.49.

Appendix Figure S6

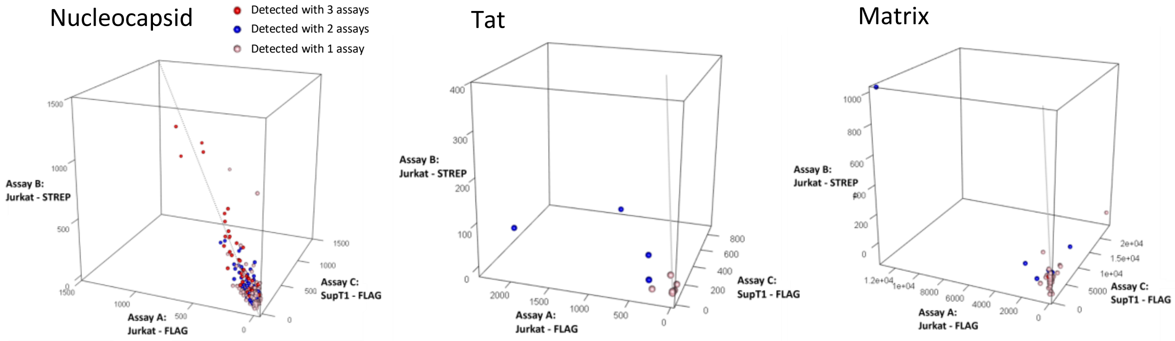

Appendix Figure S6: Interaction scores of interactions between HIV-1 proteins (Tat, Matrix and Nucleocapsid) and host RNA interactors. 3D-scatterplots of the interaction scores of each of the identified RNA interactors for assay A, B and C. Grey line depicts the xyz-diagonal. Red, blue and white dots represent RNA interactors detected by three, two or one assay respectively.

Appendix Figure S7

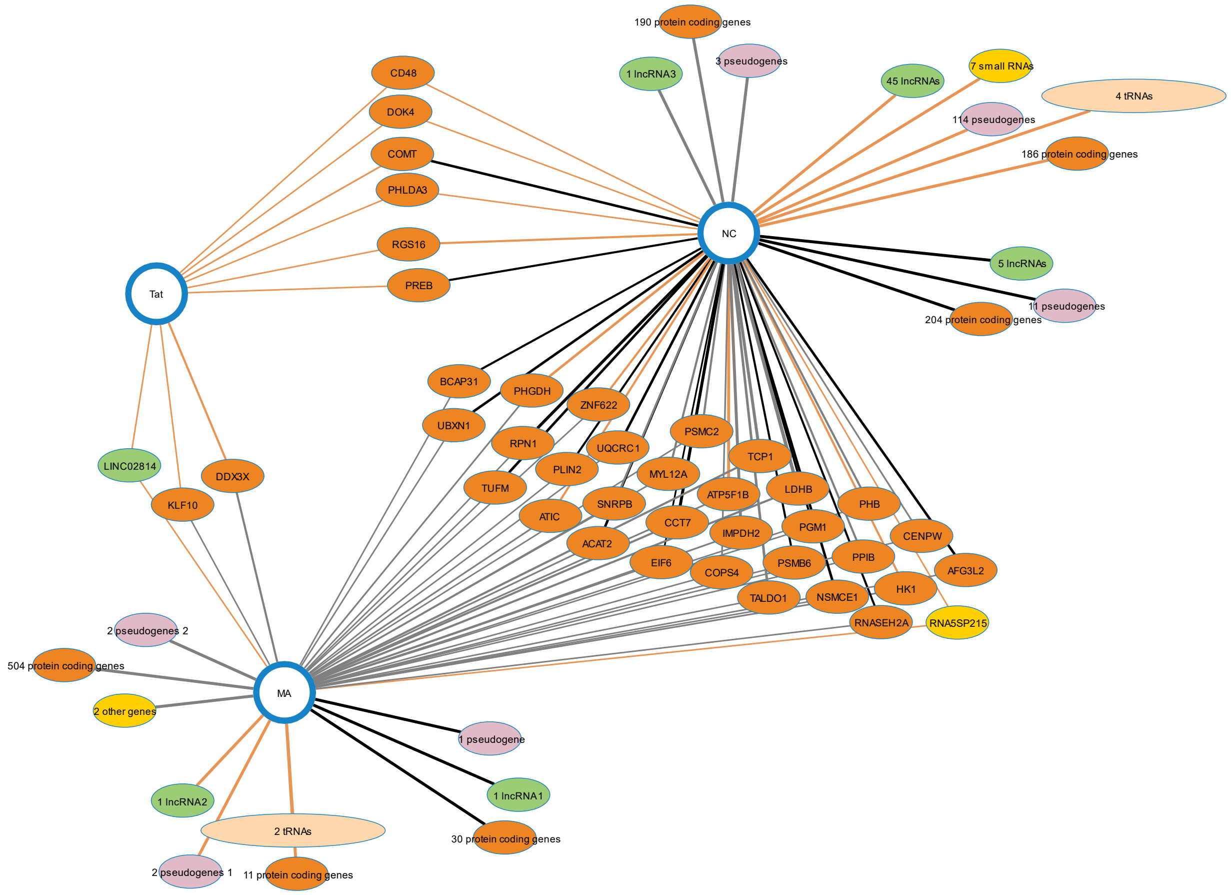

Appendix Figure S7: Interactions between HIV-1 proteins (Tat, Matrix and Nucleocapsid) and host RNA interactors in the SupT1 cell line. In total 1558 interactions were detected between host RNAs and these three HIV proteins in SupT1 cells that were either uninfected (grey), infected with NL4.3 virus (orange) or detected in both conditions(black). Thicker connectors correspond to interactions with higher interaction scores.

Appendix Figure S8

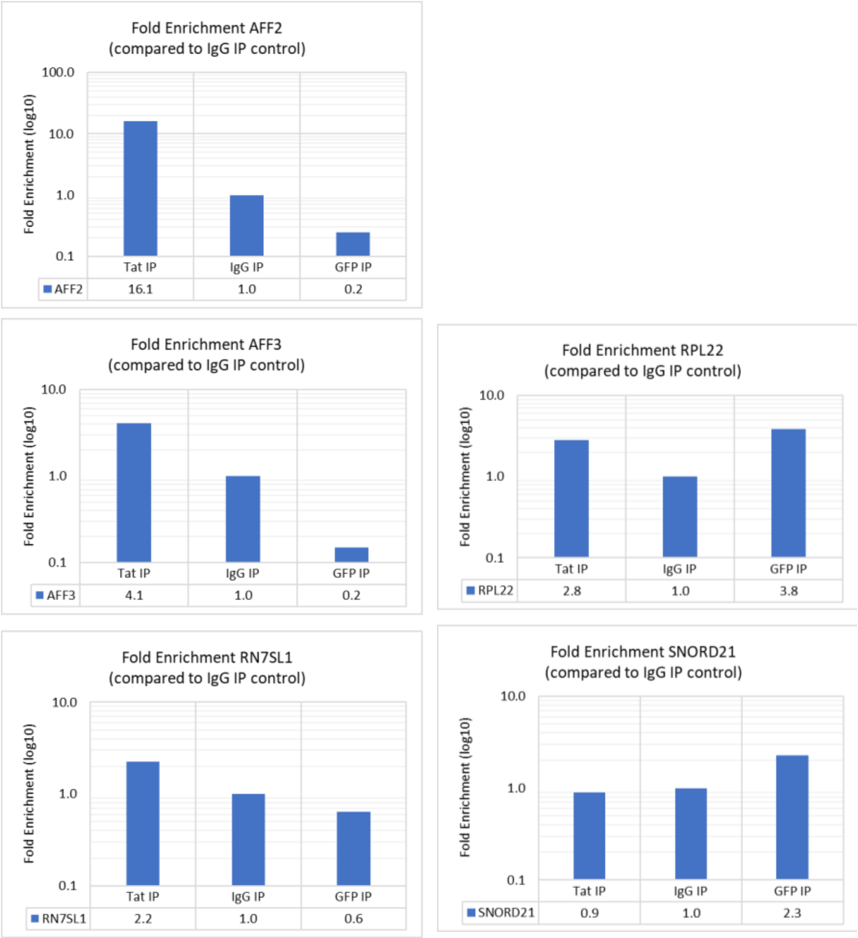

Appendix Figure S8. Fold Enrichment plots for the TAT-RNA interactors for the Jurkat cell lines with FLAG-based pulldown (Assay A). Expression is normalized to the IgG control. Results shown are from 1 TAT pulldown experiment with 1 technical replicate on which qPCR was performed to assess RNA quantities. Fold enrichments were calculated via the fold enrichment method (2^deltaCq) where raw Cq values are subtracted from the IgG control to generate the deltaCq values.

Appendix Figure S9

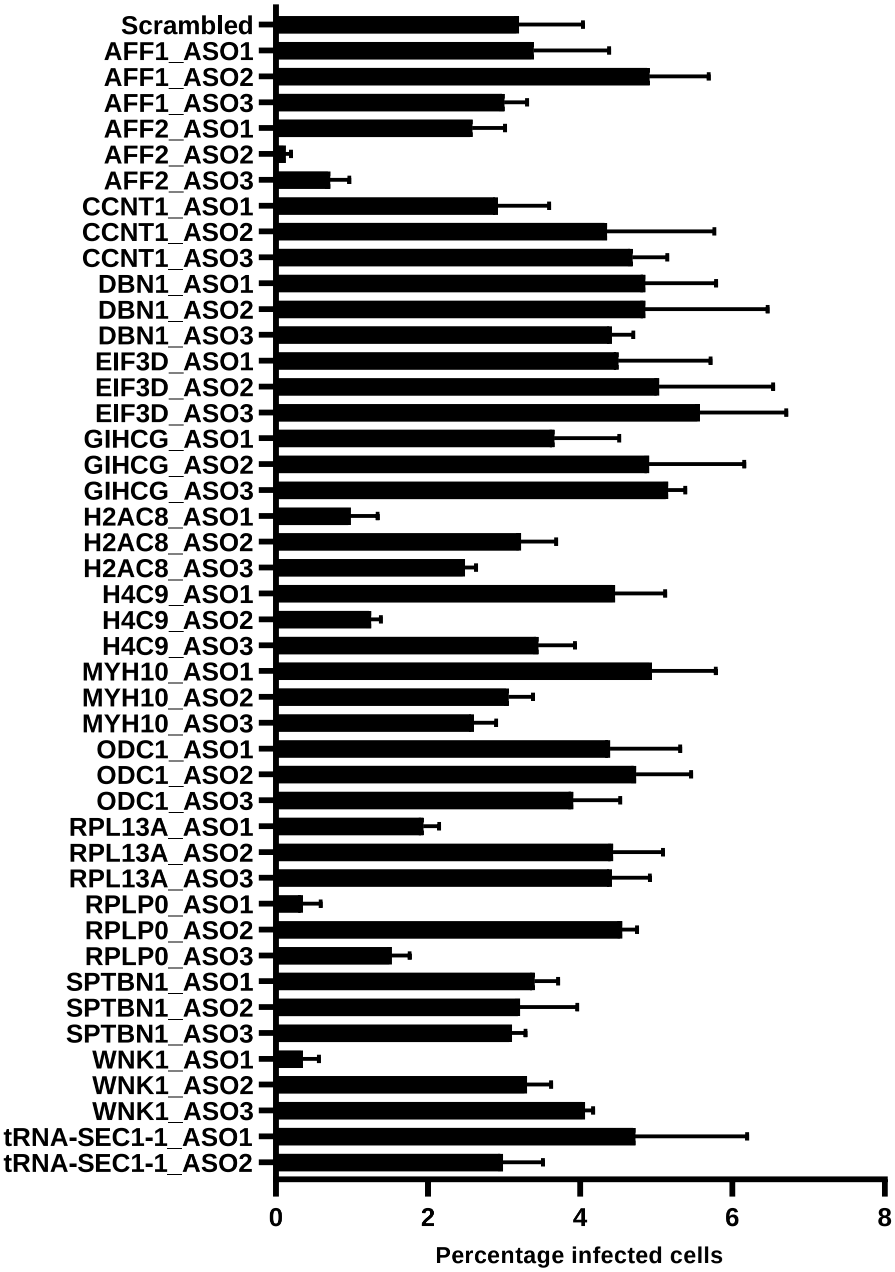

Appendix Figure S9: Antisense-oligo mediated knockdown screen on 15 host RNA interactors of HIV-1 proteins. RNA interactors were selected based on their interaction score and membership to gene ontology enrichment terms. Three ASOs were tested per RNA interactor. SupT1 cells were infected with GFP-tagged NL4.3 virus, 48h post ASO treatment. 24h post infection, the percentage of GFP+ cells was assessed by flow cytometry and compared to a control condition treated with a scrambled, non-targeting ASO. Conditions with an impaired HIV-1 infectivity were validated in an additional experiment where knockdown was confirmed with qPCR (figure 5). The experiment was performed once with 3 technical replicates for the on-target ASO treated conditions and 6 technical replicates for the scrambled control.

Appendix Figure S10

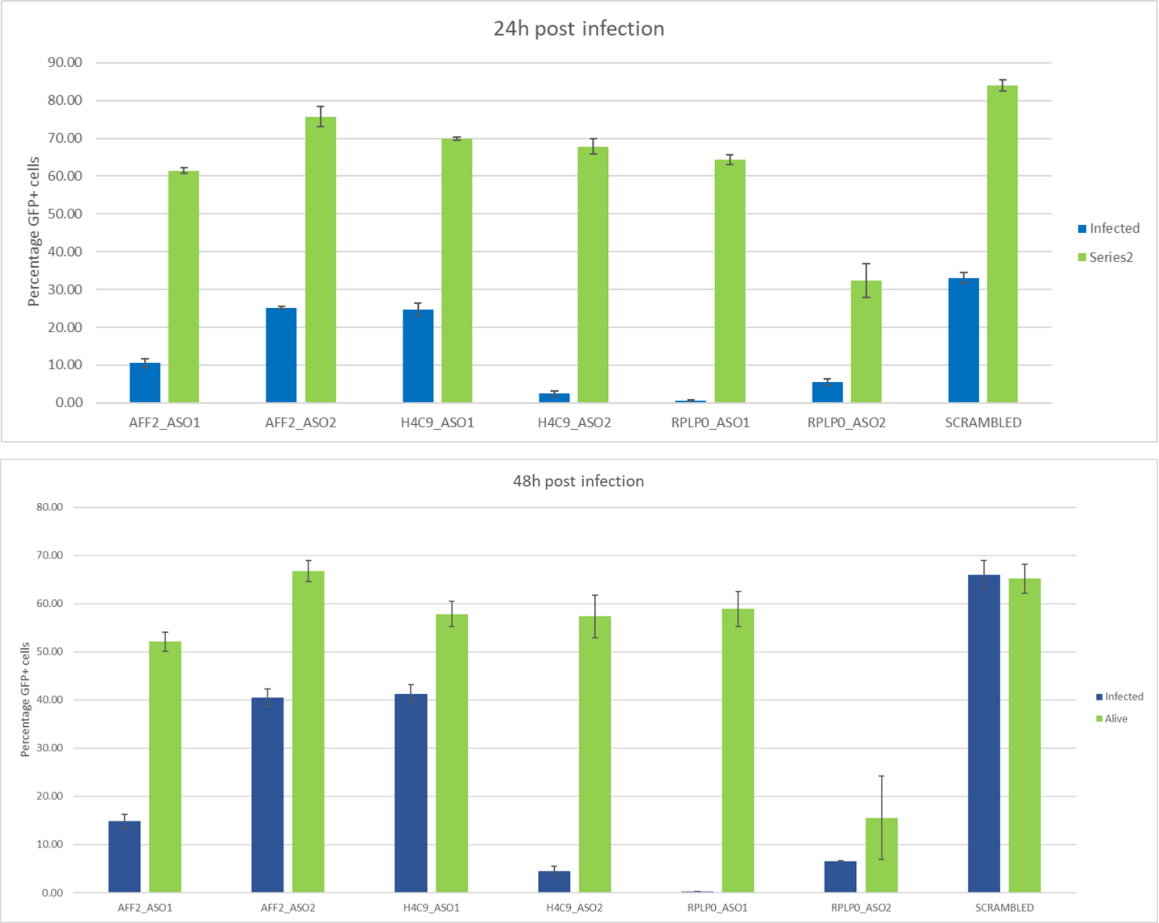

Appendix Figure S10. Cell viability and HIV-1 infection levels during ASO-mediated knockdown in SupT1 cells. Cell viability data measured via PI staining (green) and HIV-1 infection levels via EGFP (blue) on MACSQuant flow cytometer for the ASO-treated conditions versus a scrambled-ASO control in SupT1 cells. The experiment was performed once with 3 technical replicates for the on-target ASO treated conditions and 6 technical replicates for the scrambled controls.

Appendix Figure S11

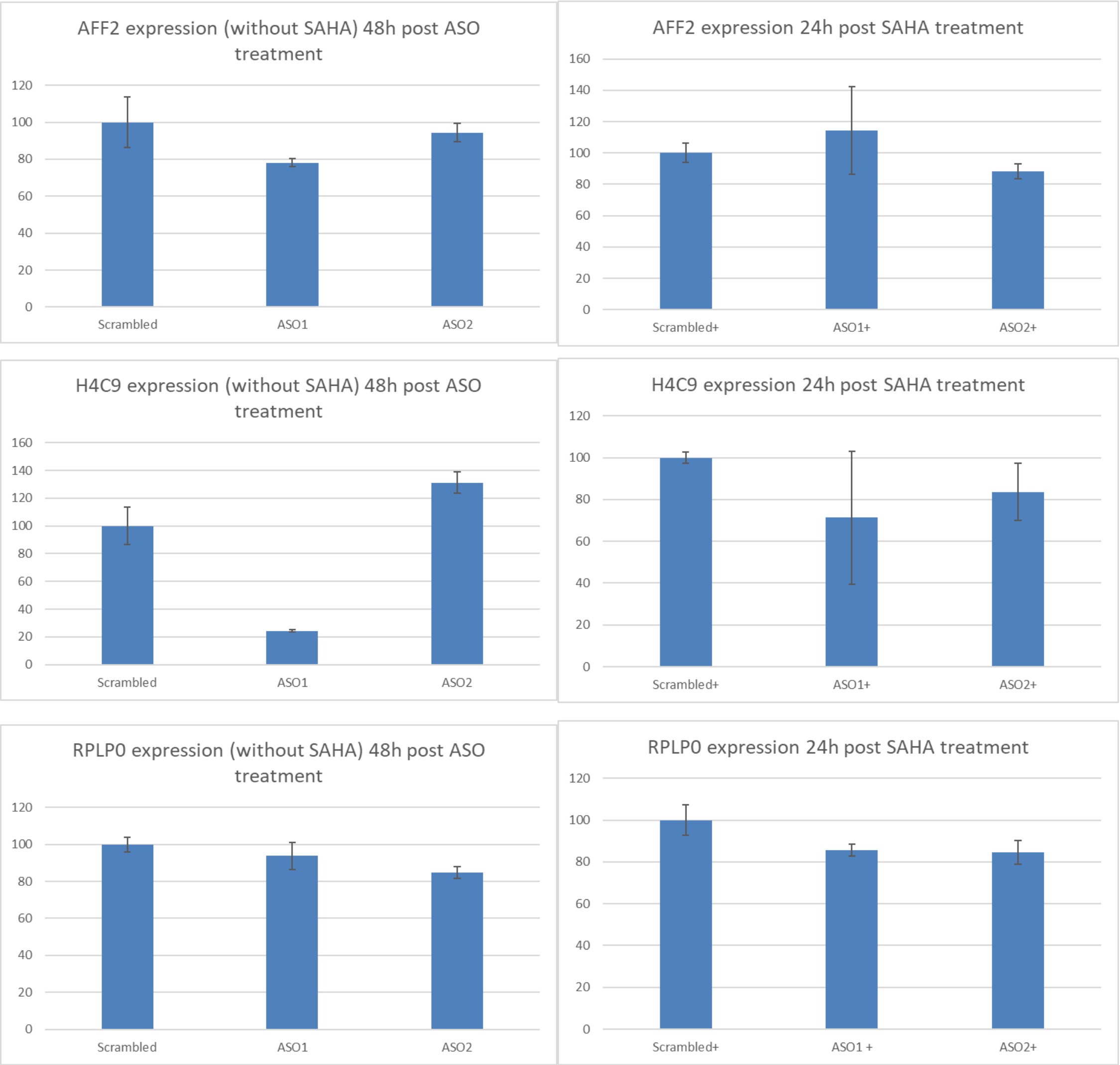

Appendix Figure S11. Knockdown assessment via qPCR for the expression of AFF2, HC49 and RPLP0 in J-Lat 10.6 after treatment with ASO. Results are normalized to the scrambled ASO control. The experiment was performed once with 3 technical replicates for each condition. Error bars represent the standard deviation, no statistical testing was performed.

Appendix Figure S12

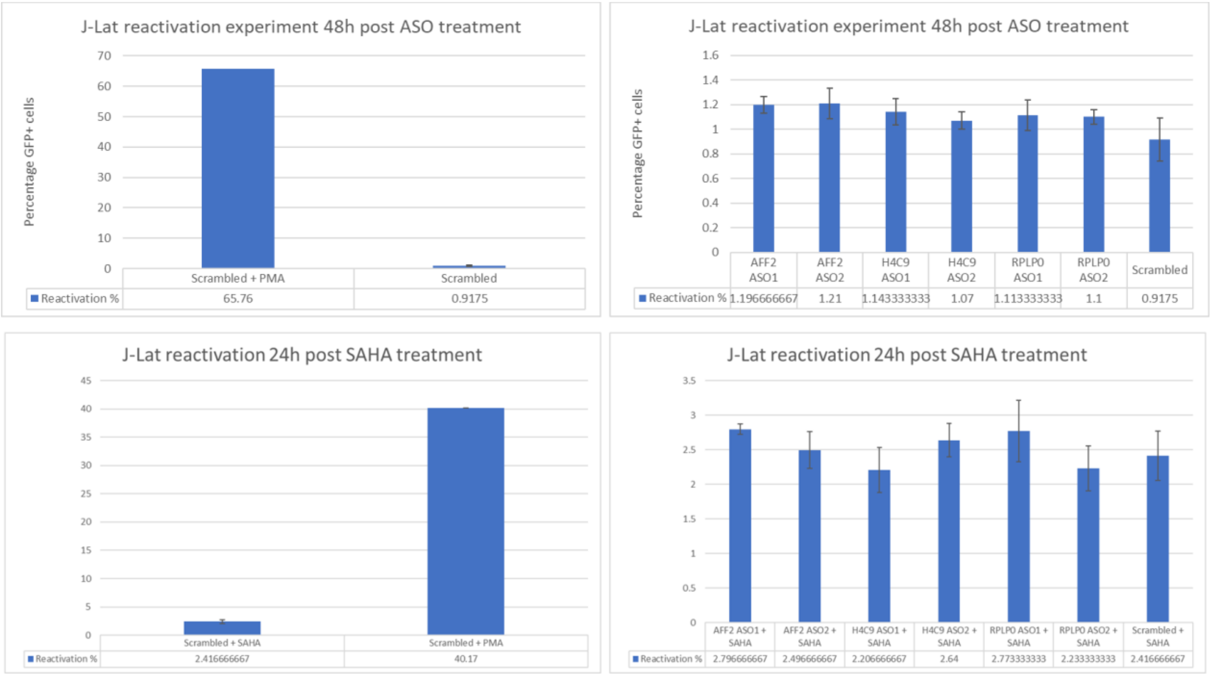

Appendix Figure S12. J-Lat reactivation measured as GFP% cells via MACSquant flowcytometer in the context without (top panels) and with SAHA (bottom panels). PMA-treated vs ASO scrambled treated controls shown on the left, ASO-treated conditions shown on the right panels. The experiment was performed once with 3 technical replicates for each condition. Error bars represent the standard deviation, no statistical testing was performed.

Appendix Table S1: The Padj and Log2(Fold change) (Log2FC) cutoffs for which an RNA molecule was considered an RNA interactor of the HIV-1 protein based on the nRIPseq results. The Padj and Log2FC values were determined with Deseq2, calculating enrichment of the RNA molecule in the immunoprecipitated sample compared to the IgG and GFP background controls. The cutoff values were determined by optimizing the signal to background proportion as described in the method section.

HIV-1 protein	nRIPseq assay	Padj cutoff	Log2FC cutoff
Nucleocapsid	Jurkat-FLAG (A)	1,00E-10	1,49
Matrix	Jurkat-FLAG (A)	1,00E-08	1,03
GP120	Jurkat-FLAG (A)	1,00E-05	1
GP160	Jurkat-FLAG (A)	1,00E-07	1,01
Capsid	Jurkat-FLAG (A)	1,00E-10	1,12
Gag	Jurkat-FLAG (A)	1,00E-06	1
GP41	Jurkat-FLAG (A)	1,00E-02	1,01
Integrase	Jurkat-FLAG (A)	1,00E-08	1,12
Nef	Jurkat-FLAG (A)	5,00E-02	1,16
p6	Jurkat-FLAG (A)	1,00E-03	1
Pol	Jurkat-FLAG (A)	1,00E-05	1
Protease	Jurkat-FLAG (A)	1,00E-06	1
Rev	Jurkat-FLAG (A)	1,00E-08	1,01
Reverse transcriptase	Jurkat-FLAG (A)	1,00E-21	2
Tat	Jurkat-FLAG (A)	1,00E-02	2
Vif	Jurkat-FLAG (A)	1,00E-09	1,26
Vpr	Jurkat-FLAG (A)	1,00E-03	1,27
Vpu	Jurkat-FLAG (A)	1,00E-02	1
Gag	Jurkat-Strep (B)	1,00E-02	2
Tat	Jurkat-Strep (B)	5,00E-02	2
Matrix	Jurkat-Strep (B)	5,00E-02	1,81
Nucleocapsid	Jurkat-Strep (B)	1,00E-02	1,26
Pol	Jurkat-Strep (B)	5,00E-02	1,37
Rev	Jurkat-Strep (B)	5,00E-02	1,12
Matrix	SupT1-Uninfected (C)	1,00E-14	1,18
Nucleocapsid	SupT1-Uninfected (C)	1,00E-21	1,89
Tat	SupT1-Uninfected (C)	1,00E-07	1
Matrix	SupT1_Infected (D)	1,00E-02	1,39
Nucleocapsid	SupT1_Infected (D)	1,00E-16	2
Tat	SupT1_Infected (D)	1,00E-04	1

Appendix Table S2: Number of identified RNA interactor biotypes per HIV-1 protein and per performed assay. Assay A: anti-FLAG nRIPseq on Jurkat cells; Assay B: anti-Strep nRIPseq on Jurkat cells; Assay C: anti-FLAG nRIPseq on SupT1 cells; Assay D: anti-FLAG nRIPseq on HIV-1 infected SupT1 cells.

Protein	Assay	lncRNA	mRNA	rRNA	tRNA	pseudogene	small ncRNA	other RNA
NC	A	12	803	0	0	42	0	0
NC	B	8	357	0	15	18	12	11
NC	C	6	421	0	0	14	0	0
NC	D	50	415	2	4	126	3	2
MA	A	1	47	0	1	0	0	0
MA	B	2	31	0	3	1	7	0
MA	C	1	566	0	0	3	0	2
MA	D	3	41	0	2	4	0	0
Rev	A	1	279	0	0	6	1	2
Rev	B	1	6	0	26	0	3	4
Tat	A	0	5	0	0	0	0	1
Tat	B	1	4	0	0	0	0	0
Tat	C	0	21	0	0	0	0	0
Tat	D	2	155	0	0	1	0	0
Gag	A	1	67	0	3	2	0	0
Gag	B	0	1	0	21	0	0	0
Pol	A	1	85	0	0	0	0	0
Pol	B	0	1	0	0	0	0	0
IN	A	0	31	0	0	4	0	0
Vpu	A	2	25	0	0	0	0	0
GP120	A	2	14	0	0	0	0	0
PR	A	1	8	0	0	0	0	0
GP41	A	0	4	0	0	0	0	0
GP160	A	0	4	0	0	0	0	0
p6	A	0	4	0	0	0	0	0
Vpr	A	0	2	0	0	0	0	0
Nef	A	0	2	0	0	0	0	0
Vif	A	0	2	0	0	0	0	0
CA	A	0	2	0	0	0	0	0

Appendix Table S3: P-values of Fisher exact tests used to assess overlap in the detected RNA interaction partners of individual HIV-1 proteins by up to four different nRIPseq assays. Assay A: anti-FLAG nRIPseq on Jurkat cells; Assay B: anti-Strep nRIPseq on Jurkat cells; Assay C: anti-FLAG nRIPseq on SupT1 cells; Assay D: anti-FLAG nRIPseq on HIV-1 infected SupT1 cells.

**Nucleocapsid**	
Overlap	p-value
Assay A - B	2.80E-267
Assay A - C	0
Assay A - D	1.80E-241
Assay B - C	1.80E-166
Assay B - D	1.60E-142
Assay C - D	6.30E-276
**Matrix**	
Overlap	p-value
Assay A - B	1.80E-28
Assay A - C	5.30E-22
Assay A - D	4.80E-30
Assay B - C	1.80E-02
Assay B - D	8.40E-02
Assay C - D	1.70E-40
**Tat**	
Overlap	p-value
Assay A - B	1
Assay A - C	5.50E-12
Assay A - D	2.30E-08
Assay B - C	1
Assay B - D	1
Assay C - D	1.20E-37
**Rev**	
Overlap	p-value
Assay A - B	0.011
**Pol**	
Overlap	p-value
Assay A - B	3.4e-03
**Gag**	
Overlap	p-value
Assay A - B	3.5e-05

Appendix Table S4: Primers and antisense oligos used in quantitative PCR assays.

Gene	Forward primer (5′-3′)	Reverse primer (5′-3′)
AFF2	TGT GCA AGG CTG TCC TTT TG	CTGGCAGTTATGGACCCACT
H4C9	TATCACCAAGCCAGCCATTC	TCCAGGAACACCTTCAACAC
RPLP0	CTTGTCTGTGGAGACGGATTAC	CCACAAAGGCAGATGGATCA
GAPDH	AGCCTCAAGATCATCAGCAATGCC	TGTGGTCATGAGTCCTTCCACGAT
ACTB	TTCCTTCCTGGGCATGGAGT	TACAGGTCTTTGCGGATGTC
PLOD1	CAACAACAAGGACAACCGCATCCA	GAATTTGTGCCACTCCCGCTCAAA

Appendix Table S5: Correlation between the interaction scores of the detected RNA interaction partners of individual HIV-1 proteins by up to four different nRIPseq assays. Assay A: anti-FLAG nRIPseq on Jurkat cells; Assay B: anti-Strep nRIPseq on Jurkat cells; Assay C: anti-FLAG nRIPseq on SupT1 cells; Assay D: anti-FLAG nRIPseq on HIV-1 infected SupT1 cells.

**Nucleocapsid**		
Overlap	Correlation	p-value
Assay A - B	0.920	0
Assay A - C	0.967	0
Assay A - D	0.901	0
Assay B - C	0.878	0
Assay B - D	0.839	0
Assay C - D	0.943	0
**Matrix**		
Overlap	Correlation	p-value
Assay A - B	0.913	3.78E-208
Assay A - C	0.123	4.66E-03
Assay A - D	0.215	5.69E-07
Assay B - C	-0.079	6.88E-02
Assay B - D	-0.046	2.94E-01
Assay C - D	0.948	1.61E-264
**Tat**		
Overlap	Correlation	p-value
Assay A - B	0.316	0.000179
Assay A - C	0.699	2.79E-21
Assay A - D	0.406	9.08E-07
Assay B - C	-0.069	4.25E-01
Assay B - D	-0.064	4.57E-01
Assay C - D	0.825	5.20E-35
**Rev**		
Overlap	Correlation	p-value
Assay A - B	0.885	1.03E-33
**Pol**		
Overlap	Correlation	p-value
Assay A - B	0.316	6.04E-01
**Gag**		
Overlap	Correlation	p-value
Assay A - B	0.289	2.41E-02

Appendix Table S6: Gene ontology enrichment analysis results. The identified RNA interaction partners per protein were used as input for the webtool DAVID 2021 [19] Gene ontology enrichment analysis with GO BP_DIRECT, CC_DIRECT and MF_DIRECT. P-value is Bonferroni-adjusted, with a cutoff of 0.

Protein	Term	Fold enrichment	p-value
Gag	GO:0002181~cytoplasmic translation	172	1.41E-114
Gag	GO:0022626~cytosolic ribosome	198	5.76E-111
Gag	GO:0003735~structural constituent of ribosome	79	2.95E-92
Gag	GO:0006412~translation	69	9.10E-89
Gag	GO:0005840~ribosome	79	3.67E-75
Gag	GO:0022625~cytosolic large ribosomal subunit	168	1.69E-65
Gag	GO:0003723~RNA binding	10	2.15E-43
Gag	GO:0022627~cytosolic small ribosomal subunit	133	8.32E-35
Gag	GO:0042788~polysomal ribosome	148	6.17E-28
Gag	GO:0005925~focal adhesion	20	1.41E-26
Gag	GO:0016020~membrane	6	3.95E-25
Gag	GO:0005829~cytosol	3	1.96E-23
Gag	GO:0015935~small ribosomal subunit	126	4.39E-19
Gag	GO:0005737~cytoplasm	3	2.60E-16
Gag	GO:0070062~extracellular exosome	4	1.14E-11
Gag	GO:0014069~postsynaptic density	16	2.14E-10
Gag	GO:0006364~rRNA processing	24	3.54E-09
Gag	GO:0005730~nucleolus	5	8.23E-08
Gag	GO:1990904~ribonucleoprotein complex	19	2.12E-07
Gag	GO:1990948~ubiquitin ligase inhibitor activity	156	1.24E-06
Gag	GO:1904667~negative regulation of ubiquitin protein ligase activity	112	1.50E-05
Gag	GO:0019843~rRNA binding	41	2.23E-05
Gag	GO:0015934~large ribosomal subunit	85	2.39E-05
Gag	GO:0045202~synapse	8	3.41E-05
Gag	GO:0042274~ribosomal small subunit biogenesis	73	9.95E-05
Gag	GO:0048027~mRNA 5-UTR binding	54	0.000140346
Gag	GO:0000027~ribosomal large subunit assembly	61	0.000216106
Gag	GO:0003729~mRNA binding	9	0.001655773
Gag	GO:0000028~ribosomal small subunit assembly	78	0.002932685
Gag	GO:0005654~nucleoplasm	2	0.003256638
Gag	GO:0006413~translational initiation	28	0.005519014
Gag	GO:0098556~cytoplasmic side of rough endoplasmic reticulum membrane	153	0.012409775

Gag	GO:0042273~ribosomal large subunit biogenesis	40	0.022546126
Gag	GO:0070181~small ribosomal subunit rRNA binding	94	0.030463889
Gag	GO:0006417~regulation of translation	17	0.036252842
IN	GO:0022626~cytosolic ribosome	48	8.96E-06
IN	GO:0002181~cytoplasmic translation	40	4.98E-05
IN	GO:0022625~cytosolic large ribosomal subunit	52	0.00016454
IN	GO:0003735~structural constituent of ribosome	19	0.00092039
IN	GO:0006412~translation	16	0.004279308
IN	GO:0005840~ribosome	18	0.011171434
IN	GO:0005829~cytosol	2	0.028570816
IN	GO:0005925~focal adhesion	9	0.033915246
IN	GO:0005737~cytoplasm	2	0.035623197
Matrix	GO:0030527~structural constituent of chromatin	93	1.34E-51
Matrix	GO:0000786~nucleosome	68	4.08E-48
Matrix	GO:0046982~protein heterodimerization activity	26	1.06E-34
Matrix	GO:0006334~nucleosome assembly	54	6.98E-32
Matrix	GO:0070062~extracellular exosome	5	1.03E-19
Matrix	GO:0032200~telomere organization	124	1.66E-18
Matrix	GO:0003677~DNA binding	7	3.00E-18
Matrix	GO:0006335~DNA replication-dependent nucleosome assembly	105	1.62E-17
Matrix	GO:0000228~nuclear chromosome	66	2.66E-15
Matrix	GO:0043505~CENP-A containing nucleosome	148	6.00E-14
Matrix	GO:0061644~protein localization to CENP-A containing chromatin	140	1.95E-13
Matrix	GO:0045296~cadherin binding	15	8.02E-12
Matrix	GO:0005634~nucleus	2	1.10E-09
Matrix	GO:0006342~chromatin silencing	50	5.96E-08
Matrix	GO:0060968~regulation of gene silencing	152	6.30E-08
Matrix	GO:0051015~actin filament binding	15	3.13E-07
Matrix	GO:0045653~negative regulation of megakaryocyte differentiation	84	2.06E-06
Matrix	GO:0040029~regulation of gene expression. epigenetic	64	8.60E-06
Matrix	GO:0006336~DNA replication-independent nucleosome assembly	62	1.05E-05
Matrix	GO:0002227~innate immune response in mucosa	60	1.28E-05
Matrix	GO:0032991~macromolecular complex	6	6.14E-05
Matrix	GO:0006352~DNA-templated transcription. initiation	43	7.27E-05

Matrix	GO:0003779~actin binding	9	0.000161012
Matrix	GO:0015629~actin cytoskeleton	10	0.000296501
Matrix	GO:0019731~antibacterial humoral response	28	0.000598265
Matrix	GO:0019904~protein domain specific binding	9	0.002506714
Matrix	GO:0003723~RNA binding	3	0.006516463
Matrix	GO:0061844~antimicrobial humoral immune response mediated by antimicrobial peptide	15	0.012113783
Matrix	GO:0001725~stress fiber	20	0.013671355
Matrix	GO:0050830~defense response to Gram-positive bacterium	13	0.026827332
Matrix	GO:0005654~nucleoplasm	2	0.027516317
Matrix	GO:0005884~actin filament	16	0.035943147
Matrix	GO:0000781~chromosome. telomeric region	10	0.036545767
Nucleocapsid	GO:0003735~structural constituent of ribosome	10	1.17E-57
Nucleocapsid	GO:0006412~translation	9	5.11E-54
Nucleocapsid	GO:0002181~cytoplasmic translation	15	2.26E-52
Nucleocapsid	GO:0022626~cytosolic ribosome	15	2.98E-49
Nucleocapsid	GO:0005840~ribosome	9	4.36E-48
Nucleocapsid	GO:0005743~mitochondrial inner membrane	5	6.38E-40
Nucleocapsid	GO:0005515~protein binding	1	1.04E-37
Nucleocapsid	GO:0005739~mitochondrion	3	2.31E-35
Nucleocapsid	GO:0022625~cytosolic large ribosomal subunit	13	2.78E-29
Nucleocapsid	GO:0005829~cytosol	2	1.70E-27
Nucleocapsid	GO:0022627~cytosolic small ribosomal subunit	14	2.45E-22
Nucleocapsid	GO:0003723~RNA binding	2	6.32E-19
Nucleocapsid	GO:0005654~nucleoplasm	2	4.64E-16
Nucleocapsid	GO:0005762~mitochondrial large ribosomal subunit	9	7.06E-13
Nucleocapsid	GO:0016020~membrane	2	8.47E-13
Nucleocapsid	GO:0032543~mitochondrial translation	7	2.83E-12
Nucleocapsid	GO:0015935~small ribosomal subunit	13	5.15E-12
Nucleocapsid	GO:0005737~cytoplasm	1	1.05E-11
Nucleocapsid	GO:0042776~mitochondrial ATP synthesis coupled proton transport	8	1.02E-10
Nucleocapsid	GO:0032200~telomere organization	13	1.70E-10
Nucleocapsid	GO:0042788~polysomal ribosome	11	1.45E-09
Nucleocapsid	GO:0030527~structural constituent of chromatin	6	2.98E-09
Nucleocapsid	GO:0006335~DNA replication-dependent nucleosome assembly	11	4.47E-09
Nucleocapsid	GO:0043505~CENP-A containing nucleosome	15	1.92E-08
Nucleocapsid	GO:0009060~aerobic respiration	7	5.23E-08
Nucleocapsid	GO:0061644~protein localization to CENP-A containing chromatin	15	9.88E-08

Nucleocapsid	GO:0070062~extracellular exosome	2	3.24E-07
Nucleocapsid	GO:0032981~mitochondrial respiratory chain complex I assembly	7	5.35E-07
Nucleocapsid	GO:0000228~nuclear chromosome	7	5.58E-07
Nucleocapsid	GO:0000786~nucleosome	4	1.41E-06
Nucleocapsid	GO:0005747~mitochondrial respiratory chain complex I	7	1.55E-06
Nucleocapsid	GO:0006626~protein targeting to mitochondrion	10	2.88E-06
Nucleocapsid	GO:0005758~mitochondrial intermembrane space	5	7.80E-06
Nucleocapsid	GO:0000028~ribosomal small subunit assembly	15	8.18E-06
Nucleocapsid	GO:0045653~negative regulation of megakaryocyte differentiation	12	1.14E-05
Nucleocapsid	GO:0005759~mitochondrial matrix	3	2.67E-05
Nucleocapsid	GO:0006120~mitochondrial electron transport. NADH to ubiquinone	7	3.10E-05
Nucleocapsid	GO:0008137~NADH dehydrogenase (ubiquinone) activity	7	4.63E-05
Nucleocapsid	GO:0005753~mitochondrial proton-transporting ATP synthase complex	11	7.51E-05
Nucleocapsid	GO:0005634~nucleus	1	9.87E-05
Nucleocapsid	GO:0005783~endoplasmic reticulum	2	9.93E-05
Nucleocapsid	GO:0006122~mitochondrial electron transport. ubiquinol to cytochrome c	14	0.000110129
Nucleocapsid	GO:1902600~hydrogen ion transmembrane transport	4	0.000260189
Nucleocapsid	GO:0046982~protein heterodimerization activity	2	0.000355419
Nucleocapsid	GO:0006336~DNA replication-independent nucleosome assembly	9	0.000389933
Nucleocapsid	GO:0005750~mitochondrial respiratory chain complex III	13	0.00055587
Nucleocapsid	GO:0005925~focal adhesion	2	0.000708923
Nucleocapsid	GO:0019843~rRNA binding	6	0.001865036
Nucleocapsid	GO:0006352~DNA-templated transcription. initiation	7	0.002175727
Nucleocapsid	GO:0045333~cellular respiration	6	0.003772865
Nucleocapsid	GO:0015934~large ribosomal subunit	10	0.004398549
Nucleocapsid	GO:0015986~ATP synthesis coupled proton transport	8	0.004681906
Nucleocapsid	GO:0005761~mitochondrial ribosome	8	0.005160778
Nucleocapsid	GO:0006334~nucleosome assembly	4	0.00712493
Nucleocapsid	GO:0017018~myosin phosphatase activity	4	0.009801245

Nucleocapsid	GO:0008121~ubiquinol-cytochrome-c reductase activity	16	0.010167762
Nucleocapsid	GO:0015078~hydrogen ion transmembrane transporter activity	6	0.013675379
Nucleocapsid	GO:1990948~ubiquitin ligase inhibitor activity	14	0.021854674
Nucleocapsid	GO:1904667~negative regulation of ubiquitin protein ligase activity	12	0.030019108
Nucleocapsid	GO:0032991~macromolecular complex	2	0.042230028
Pol	GO:0005829~cytosol	2	6.66E-07
Pol	GO:0005739~mitochondrion	4	1.02E-06
Pol	GO:0005743~mitochondrial inner membrane	5	0.020364807
Pol	GO:0005654~nucleoplasm	2	0.030876085
Pol	GO:0005759~mitochondrial matrix	6	0.03475978
Pol	GO:0003723~RNA binding	3	0.03899929
Protease	GO:0090263~positive regulation of canonical Wnt signaling pathway	76	0.048086373
Rev	GO:0002181~cytoplasmic translation	22	1.01E-25
Rev	GO:0022626~cytosolic ribosome	25	2.21E-25
Rev	GO:0006412~translation	11	2.46E-22
Rev	GO:0003735~structural constituent of ribosome	12	2.47E-22
Rev	GO:0005840~ribosome	13	9.46E-22
Rev	GO:0005739~mitochondrion	3	1.60E-15
Rev	GO:0016020~membrane	3	4.39E-14
Rev	GO:0003723~RNA binding	3	1.09E-13
Rev	GO:0022627~cytosolic small ribosomal subunit	22	1.25E-11
Rev	GO:0005515~protein binding	1	2.33E-11
Rev	GO:0022625~cytosolic large ribosomal subunit	18	2.38E-11
Rev	GO:0070062~extracellular exosome	2	8.27E-11
Rev	GO:0005743~mitochondrial inner membrane	5	4.55E-09
Rev	GO:0005829~cytosol	2	1.65E-08
Rev	GO:0015935~small ribosomal subunit	25	4.73E-08
Rev	GO:0005654~nucleoplasm	2	3.61E-07
Rev	GO:0005925~focal adhesion	4	0.000183239
Rev	GO:0005759~mitochondrial matrix	4	0.000199854
Rev	GO:0005737~cytoplasm	1	0.000291706
Rev	GO:0030527~structural constituent of chromatin	8	0.000867082
Rev	GO:0019843~rRNA binding	13	0.000912257
Rev	GO:0000786~nucleosome	6	0.001398232
Rev	GO:0032200~telomere organization	18	0.002232314
Rev	GO:0005634~nucleus	1	0.002363591
Rev	GO:0006335~DNA replication-dependent nucleosome assembly	16	0.00642987
Rev	GO:0042788~polysomal ribosome	13	0.031661399

Rev	GO:0000228~nuclear chromosome	9	0.036526312
Rev	GO:0005783~endoplasmic reticulum	2	0.040197544
Tat	GO:0032783~ELL-EAF complex	731	0.000192621
Tat	GO:0010468~regulation of gene expression	25	0.019072677

Appendix Table S7: Gene ontology enrichment analysis results. The uniquely identified RNA interaction partners per protein for nRIPseq assay D in HIV-1 infected SupT1 cells were used as input for the webtool DAVID 2021 [19] Gene ontology enrichment analysis with GO BP_DIRECT, CC_DIRECT and MF_DIRECT. P-value is Bonferroni-adjusted, with a cutoff of 0.05.

HIV-1 protein	Category	Term	Fold Enrichment	Bonferroni
TAT	GOTERM_MF_DIRECT	GO:0005515~protein binding	1.24826631	0.006328938
MA	GOTERM_CC_DIRECT	GO:0015629~actin cytoskeleton	39.31226054	0.000189992
MA	GOTERM_CC_DIRECT	GO:0016459~myosin complex	128.25625	0.011764858
MA	GOTERM_CC_DIRECT	GO:0005886~plasma membrane	3.042965709	0.108515049
MA	GOTERM_MF_DIRECT	GO:0003779~actin binding	40.39144385	1.98326E-09
MA	GOTERM_MF_DIRECT	GO:0051015~actin filament binding	39.9217759	9.75794E-05
NC	GOTERM_BP_DIRECT	GO:0043567~regulation of insulin-like growth factor receptor signaling pathway	54.08988764	0.038230388
NC	GOTERM_CC_DIRECT	GO:0070062~extracellular exosome	2.215420823	0.000120146
NC	GOTERM_CC_DIRECT	GO:0005829~cytosol	1.514344362	0.010804534
NC	GOTERM_CC_DIRECT	GO:0005739~mitochondrion	2.242123863	0.021270434
NC	KEGG_PATHWAY	hsa03010:Ribosome	5.661437908	0.033271568

Appendix Table S8: Used antisense oligonucleotides (ASOs).

Antisense oligo (ASO)	Sequence
RPL13A ASO1	CCTTGTCGTAGGGCGG
RPL13A ASO2	GGTAAGGCCGCTGCCT
RPL13A ASO3	GTGCAGCGTAAGCTGG
H4C9 ASO1	ACACTCCGCGGGTCTC
H4C9 ASO2	TGGATGTTGTCGCGCA
H4C9 ASO3	GCCGAATGGCTGGCTT
GIHCG ASO1	ACTCAGATCCCCGGGG
GIHCG ASO2	AACCAGGCCATTGGGC
GIHCG ASO3	GCGACAGTGAGGAGCC
ODC1 ASO1	ACTGAAGGCGCCAAGG
ODC1 ASO2	TTCTGAGCGTGGCACC
ODC1 ASO3	ACGCCGGTGATCTAAG
MYH10 ASO1	ACCTCCAGGCCTGCGT
MYH10 ASO2	GGATGTACACGCGTGC
MYH10 ASO3	TTTGTTGGCGGCTGCT
SPTBN1 ASO1	GGCGATCGCTGTCTGG
SPTBN1 ASO2	GAGAGAGCGCGCTACA
SPTBN1 ASO3	GTAAGGTGGCGGCACT
DBN1 ASO1	TAACCCAGCGCTGGGG
DBN1 ASO2	CCTAGGCGCCTGGACC
DBN1 ASO3	GGAGAAGGGCCCTTCG
H2AC8 ASO1	CCTGGAAGAACGCGTT
H2AC8 ASO2	GATCTCGGCCGTCAGA
H2AC8 ASO3	TTGCGGATGGCTAGCT
AFF2 ASO1	TGGGCATCGATGCGCA
AFF2 ASO2	GGCGCAGGTGAGTTCC
AFF2 ASO3	TGGTTAGGGCCCTGGC
AFF1 ASO1	GAACTCGGGCCGAGAC
AFF1 ASO2	TTCATACGGCCGGGCG
AFF1 ASO3	GGCAATGACCGGTCAC
CCNT1 ASO1	GCTTCGCGCGGTATTC
CCNT1 ASO2	CGGAGACTGGCAGGGA
CCNT1 ASO3	TAAGCCAGCTGGCACT
tRNA-SeC-TCA-1-1 ASO1	CGCCCGAAAGGTGGAA
tRNA-SeC-TCA-1-1 ASO2	TCTGTCGCTAGACAGC
WNK1 ASO1	CACTGAGCTCGGTGGC
WNK1 ASO2	AGCCCGGGCTACAAAG
WNK1 ASO3	CTTTTGGCCGGGCGAA
RPLP0 ASO1	TGTAATGTGCGGCCGG
RPLP0 ASO2	TTTGCGCTGGGGCAAC

RPLP0 ASO3	CGGACACCCTGGGGGA
EIF3D ASO1	TACTGCGGCAGTGTCG
EIF3D ASO2	CTTCTGTGTGCGCGCT
EIF3D ASO3	AGCGCTGTGGGGAGAT
